# Puerperal Insanity

**Published:** 1923-06

**Authors:** 


### PUERPERAL INSANITY.

DURING the Post-Graduate Course for the Inspectors of Midwives, Sir Robert Armstrong-Jones, M.D., Lord Chancellor's Visitor in Lunacy, delivered a lecture on "The Nature of Puerperal Insanity and a Few Remarks on How to Nurse a Case." He said that there are to-day over 70,000 insane women under certificates in this country, of whom at least 6 per cent. are insane from causes connected with the occasion of childbirth. Puerperal fever may be said to have disappeared from our lying-in hospitals. Statistically, puerperal infection is responsible for less than two deaths in every 1,000 births, although probably four in every 1,000 women are affected by it. Its chief cause is to be found in insanitary conditions, in overcrowding, impure air and serious drainage defects.

### A High Toll.

There are no trustworthy statistics of the incidence of puerperal insanity since the war, but pre-war figures gave its incidence as 6.4 per cent. among private patients among the well-to-do classes, and 8.1 among the poorer classes, or an average of 7.2 per cent. of all occurring insanity; that is, of all women at all ages who have become insane. About 800 mothers become victims to insanity each year at this period, which is a high toll taken of the pregnant women, whose great time of joy is thus being transformed into one of fearful anxiety, acute distress, and, too often, of irretrievable disaster.

### Temperamental Dangers.

Those who have studied the mental changes which occur under normal circumstances during a natural pregnancy must realise that in persons of a highly susceptible disposition, those that are of the quick, nervous, and sanguine temperament, these changes are apt to be very decided and definite and to become exaggerated. The ordinary digestive troubles, the various forms of neuralgia, the occasional insomnia, the feeling of weight and dizziness, are apt to develop into more than the ordinary irritability and restlessness. They sometimes proceed to fractiousness, excitement, or extreme despondency, ending in a complete mental breakdown; the normal affections may turn into suspicion and hatred, the reproductive functions experience a reversal, and the ordinary maternal instincts become so perverted that they are a real and a grave danger to both mother and child.

### The Stigma of Certification.

There is a Bill now before Parliament to make it legal for a patient suffering from a definite attack of insanity to be cared for for at least six months in her own home, or in a private asylum, or in a mental hospital, but without the certificates. This will prove an inestimable boon not only to the sufferer, but also to relatives and friends, because when she gets well, as in all probability she will, there will be no finger of scorn pointed at her suggesting that she had been a certified lunatic. To certify a mother after the birth of her baby is to remind her ever after that she had once been regarded as insane. Much better, therefore, is it to do everything one can to carry out home treatment.

